# Civilian gunshot wounds to the head: a case report, clinical management, and literature review

**DOI:** 10.1186/s41016-020-00227-9

**Published:** 2021-02-03

**Authors:** Haoyi Qi, Kunzheng Li

**Affiliations:** 1 Qinghai University, No. 251 Ningda Road, Xining, 810016 Qinghai Province China; 2 The Affiliated Hospital of Qinghai University, No. 29 Tongren Road, Xining, 810000 Qinghai Province China

**Keywords:** Gunshot wound, Head trauma, Penetrating brain injury, Traumatic brain injury

## Abstract

**Background:**

Civilian gunshot wounds to the head refer to brain injury caused by projectiles such as gun projectiles and various fragments generated by explosives in a power launch or explosion. Gunshot wounds to the head are the deadliest of all gun injuries. According to literature statistics, the survival rate of patients with gunshot wounds to the head is only 9%. Due to the strict management of various types of firearms, they rarely occur, so the injury mechanism, injury and trauma analysis, clinical management, and surgical standards are almost entirely based on military experience, and there are few related reports, especially of the head, in which an individual suffered a fatal blow more than once in a short time. We report a case with a return to almost complete recovery despite the patient suffering two gunshot injuries to the head in a short period of time.

**Case presentations:**

We present a case of a 53-year-old man who suffered two gunshot injuries to the head under unknown circumstances. On initial presentation, the patient had a Glasgow Coma Scale score of 6, was unable to communicate, and had loss of consciousness. The first bullet penetrated the right frontal area and finally reached the right occipital lobe. When the patient reflexively shielded his head with his hand, the second bullet passed through the patient’s right palm bone, entered the right frontotemporal area, and came to rest deep in the lateral sulcus. The patient had a cerebral hernia when he was admitted to the hospital and immediately entered the operating room for rescue after a computed tomography scan. After two foreign body removals and skull repair, the patient recovered completely.

**Conclusions:**

Gunshot wounds to the head have a high mortality rate and usually require aggressive management. Evaluation of most gunshot injuries requires extremely fast imaging examination upon arrival at the hospital, followed by proactive treatment against infection, seizure, and increased intracranial pressure. Surgical intervention is usually necessary, and its key points include the timing, method, and scope of the operation.

## Background

The surgical management of gunshot wounds to the head (GWH) is still a challenging issue in neurosurgery [1]. Even after experience acquired during the two World Wars and multiple local wars, the surgical management of such patients still needs further discussion because mortality and morbidity remain high despite technological improvements in the last decades [1, 2]. Neurosurgeons vary considerably in their approaches to GWH. Studies have shown that the age of the patient, a high preoperative Glasgow Coma Scale (GCS) score, lack of pupil abnormalities, and absence of intracerebral hematoma are predictors of a good prognosis [1].

The largest retrospective studies to date have shown that penetrating GWH are very often fatal even with appropriate medical and surgical treatment, with 71% of patients dying at the scene, 66-90% of those dying before reaching a hospital, and up to a 51% survival rate among those reaching the hospital alive [3–5]. We highlight the current management guidelines, prognostic factors, and survival outcomes in patients with penetrating GWH. We report a case that is unique due to the successful outcome and return to almost complete recovery despite two gunshots to the head and a low GCS score. We defined the almost complete recovery as the ability to complete simple, repetitive farming activities by himself without the care of his family.

## Case presentation

**Fig. 1:**
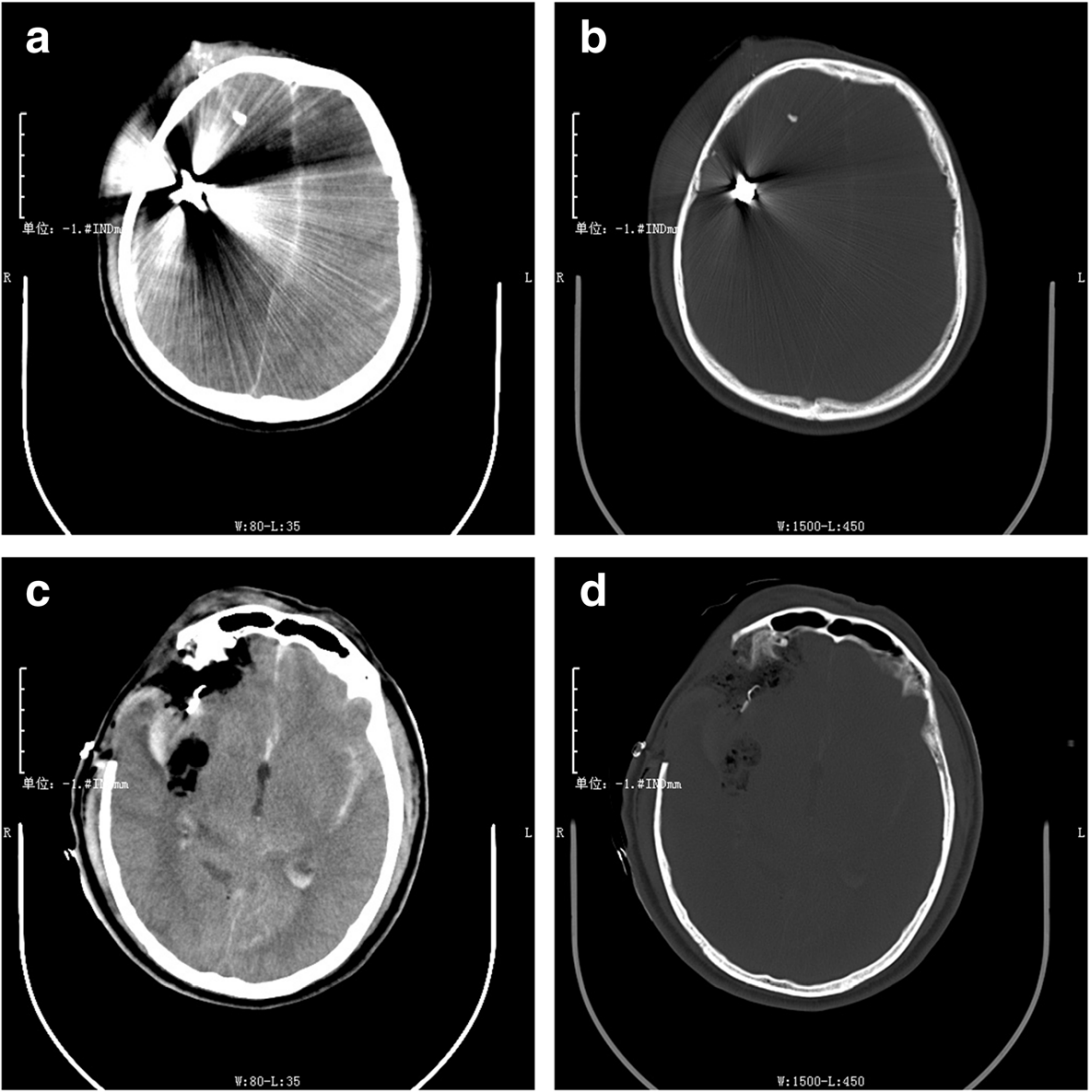
Computed tomography (CT) upon admission. **a** Soft tissue windows and **b** bone windows, showing the metal artifacts from the bullet case. Postoperative CT on the same day in soft tissue windows **c**, and bone windows **d** after decompressive craniectomy

**Fig. 2:**
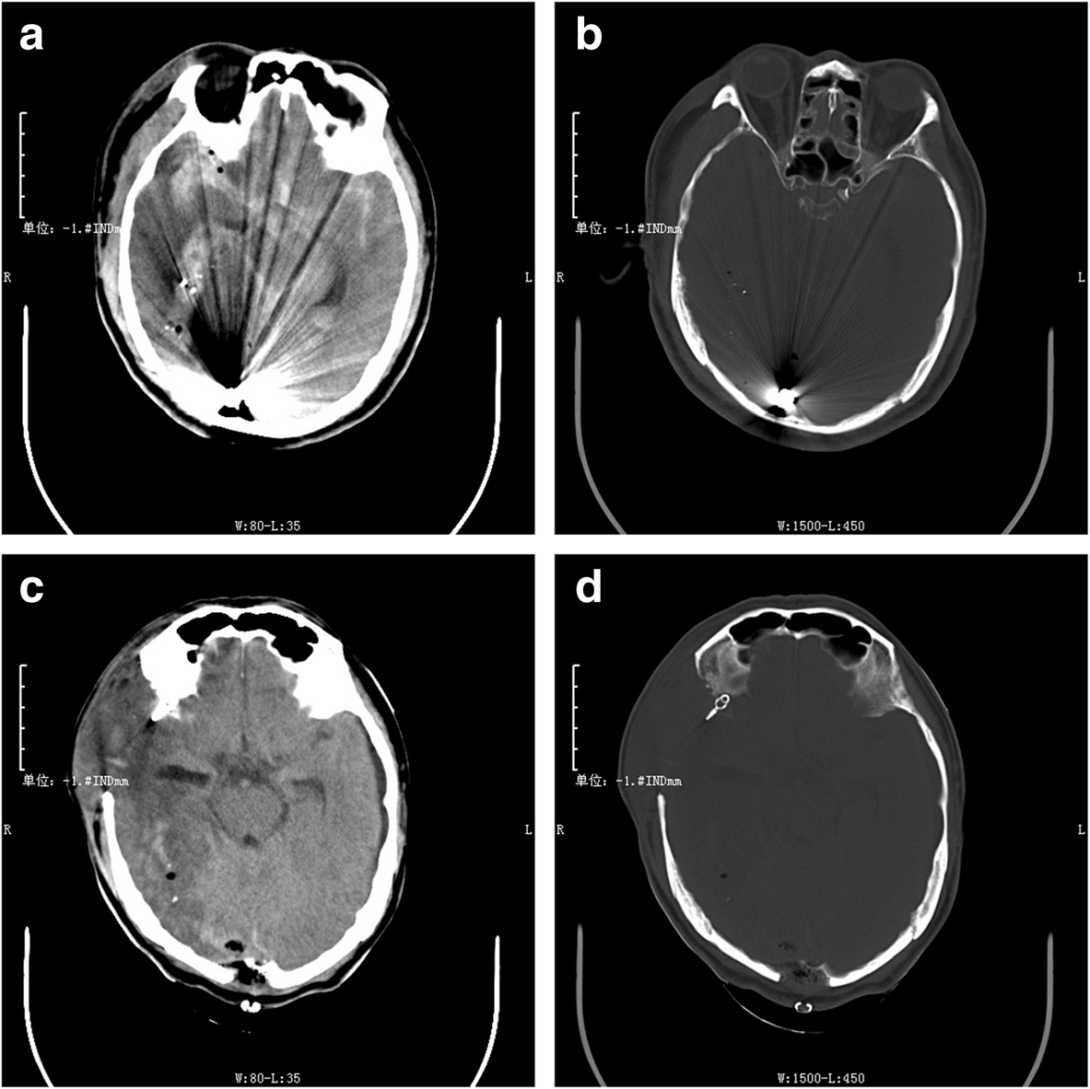
Computed tomography (CT) upon admission. **a** Soft tissue windows and **b** bone windows, showing the bullet fragment in the occipital lobe. Postoperative CT scan in soft tissue windows **c** and bone windows **d** after the second operation, clearly showing the skull window accommodating some swelling

**Fig. 3:**
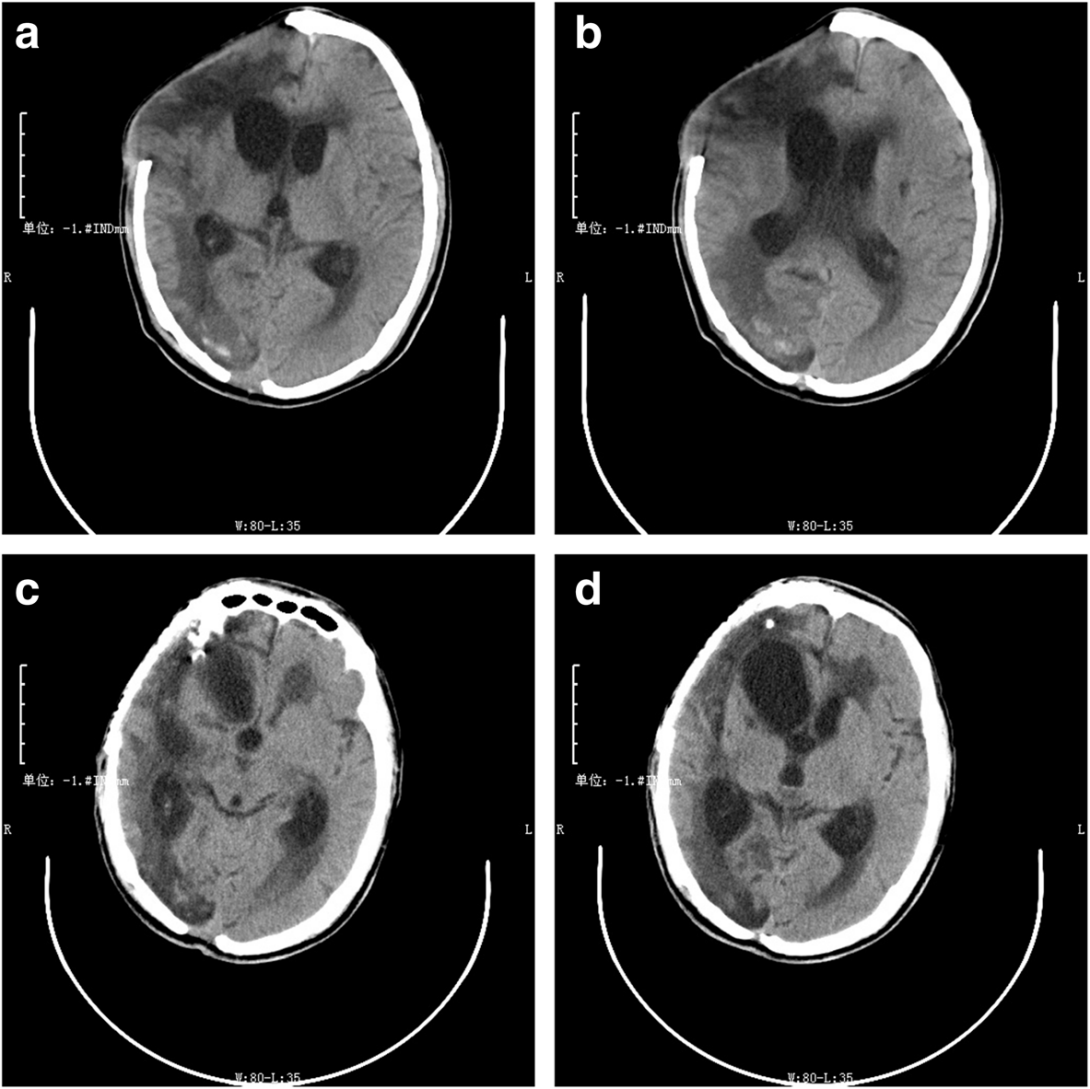
Two months after discharge, a head computed tomography (CT) scan **a** and **b** revealed hydrocephalus. **c** and **d** show the head CT scan of the patient 1 month after the repair of the skull defect

A 53-year-old male with two GWH to his right cerebral hemisphere presented with a GCS score of 6 to the hospital. According to the emergency physician’s report, the patient was taken to the hospital about 3 h after the injury. The patient was on the way to the hospital with a fixed right pupil, so he was given mannitol timely in the ambulance. We speculated that the duration of brain herniation was at least 1 h. He was hemodynamically stable and intubated, there were two adjacent bullet holes in the patient’s right frontal area, and no ballistic exit was seen. Neurological examination revealed that his right pupil was fixed and dilated, and his left pupil was 2.5-mm wide and reactive. He was responsive to pain stimuli but not to verbal stimuli. A computed tomography (CT) scan of the head revealed a bullet trajectory with a right frontal comminuted fracture and bony and metallic fragments in the right frontal and right occipital lobes. There was also some brain tissue swelling with a midline shift to the left and subarachnoid hemorrhage (Fig. 1). Since the patient had a brain herniation at admission, he was immediately transferred to the operating room for debridement and decompressive craniectomy after the first CT scan. Considering the specificity of the patient’s intracranial hematoma location and foreign body location, we performed an extended pterional approach and decompressive craniectomy in time. We did not temporarily remove the foreign body in the occipital lobe but waited for the patient’s vital signs to stabilize after the first operation and then evaluated whether it was suitable for removal or maintenance. The analysis of why this decision was taken is presented in detail in the surgical management section. The patient recovered well after the first operation with no infection or brain abscess development and underwent a second operation 2 weeks later to remove the foreign body in the occipital lobe (Fig. 2). He was discharged 7 days later. The patient visited the hospital for a re-examination 2 months after the injury, and hydrocephalus was found to have occurred. We performed skull repair 3 months after injury. The patient came in for a 6-month follow-up. During the subsequent follow-up, hydrocephalus did not continue to develop, and head CT yielded no new or concerning findings, so we did not perform additional clinical management (Fig. 3). The last telephone follow-up was performed a year after his injury. According to the patient’s family members, the self-care ability of the patient was fair, and he could complete housework alone. There was no obvious cognitive impairment, but his personality had slightly changed. The main manifestation was that he did not like to communicate with others. He did not experience seizures or other neurological symptoms. The Wisconsin card sorting test was used to assess the patient’s performance during the follow-up. The result was good. A series of non-cognitive function evaluations, such as the Functional Activity Questionnaire and Hamilton Depression Scale, were also carried out. The results were satisfactory, and the patient showed no signs of anxiety or depression.

## Discussion

### Prehospital treatment

Based on the outcomes of a prospective study, all GWH patients should initially receive aggressive resuscitation [6]. Patients with stable vital signs should be examined by CT. If the patient’s GCS score after resuscitation is 3 to 5 and no operable hematomas are present, then no further therapy should be offered [2, 6]. All patients with a GCS score greater than 5 should receive aggressive surgical therapy [2, 6]. However, some recent retrospective studies have shown that GCS score at admission and the status of pupils and hemodynamics seem to be the most significant predictors of outcome in penetrating GWH. CT scans, bi- or multilobar injury, and intraventricular hemorrhage were correlated with poor outcomes. Patients with a GCS score > 8, normal pupil reaction, and single-lobe brain injury may benefit from early aggressive management [7]. After undergoing a quick primary survey, all GWH victims with a GCS score < 9 should be intubated, oxygenated, and ventilated. Systolic blood pressure should be maintained above 90 mmHg. After secondary survey and resuscitation, the patient should undergo a CT scan of the head [1, 7, 8]. In our case, although our patient could breathe when the emergency physician arrived at the scene, they still intubated him in time, which also won us valuable time for the subsequent emergency treatment. Grahm et al. showed that preventing secondary injuries from hypoxia and hypotension by field resuscitation improved patient outcomes [6]. Although Kaufman et al. did not specify whether the GCS score was evaluated before or after resuscitation, a few patients with GCS scores of 3 to 5 clearly have potential for a reasonable recovery. Using the GCS score at admission to categorize the patients’ extent of injury and then to predict the outcome is only valid if the patient’s best score is not obscured by other conditions, such as hypoxia, hypotension, or operable hematomas. Otherwise, it is difficult to make an accurate prognosis. In addition, GCS scores obtained after resuscitation eliminate the secondary causes of a decreased level of consciousness and accurately predict the extent of the initial injury and the patient’s outcome [6]. In a 5-year retrospective review of 132 civilian patients with craniocerebral gunshot wounds, increasing survival was associated with aggressive resuscitation in all patients and resuscitation with blood products and hyperosmolar fluids were independently associated with survival. A GCS score of 3-5 and bihemispheric injury should not prevent early resuscitation, but a decision for expectant supportive care should be made when the patient has been stabilized and then reassessed, as some may improve. It is therefore the post-resuscitation GCS score that should be used for decision-making [4]. Acute traumatic coagulopathy (ATC) may develop in patients with isolated head injury (which includes GWH) and in the setting of multiple injuries with major blood loss and shock [9, 10]. This latter scenario includes GWH. The diagnosis of ATC should be predicted rapidly before admission, and treatment should be prepared as soon as possible. Massive transfusion protocols have been developed in many trauma centers; replacement of blood and clotting components should be prepared at admission [11]. However, the optimal ratio of various plasma substitutes and blood products is uncertain and remains under investigation [12].

In summary, the prehospital treatment of GWH should include early aggressive resuscitation, correction of hypotension and hypoxia, maintenance of persistent bleeding, early intervention of ATC with emergency physician experience, and an urgent CT scan.

### Imaging studies

**Fig. 4:**
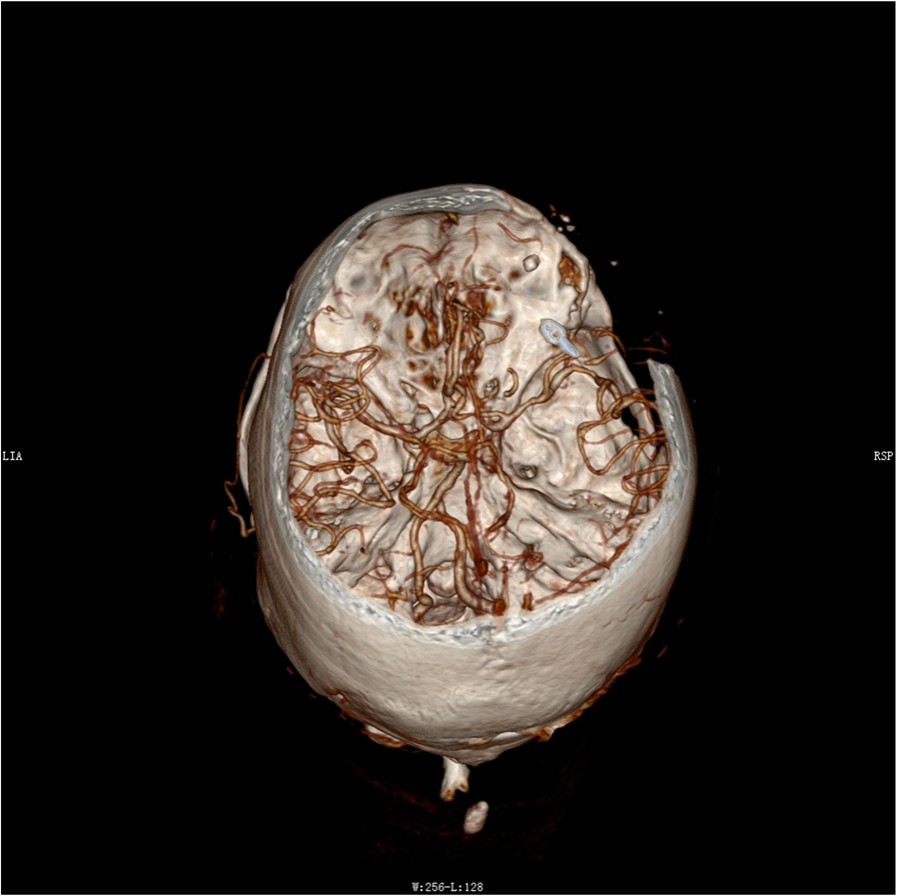
In this three-dimensional computed tomography image, the arrow demonstrates the aneurysm clip at M3 of the right middle cerebral artery. This is an unruptured aneurysm that we found by accident during the first operation. We clipped the aneurysm while exploring the deep foreign body

Neurosurgeons all over the world agree that patients with brain injury should be sent to the imaging center for head CT scans as soon as they arrive at the hospital. Plain radiographs of the head can be helpful in assessing the bullet trajectory, the presence of large foreign bodies, and the presence of intracranial air. However, when CT scanning is available, plain radiographs are not essential and are not recommended as routine [13]. No imaging technique is faster and more accurate than CT. It has almost no contraindications, except for pregnant women. A CT scan of the head defines the bullet’s trajectory, entry and exit sites, extent of intracranial fragments and proximity to major blood vessels and the ventricles, and pressure on the ambient cistern. In addition, a CT scan of the head will determine the need for surgery and define the strategy for surgical treatment. It is the recommended imaging modality with 5-mm-thick continuous slices along the Reid line from the vertex to the foramen magnum for evaluating cranial trauma [14]. Patients who are found to have risk factors on CT scans, including intracerebral hematomas, orbital and facial craniocerebral injury, and patients with projectiles penetrating two or more ventricles, anterior circulation, and trajectories close to vertebrobasilar vessels, cavernous sinus, the dural venous sinuses, and the Sylvian fissure should undergo CT angiography (CTA), and if necessary, routine digital subtraction angiography, to rule out traumatic intracranial aneurysms [13, 15]. In our case, because of the emergency situation at the time, our patient was transferred to the operating room without CTA imaging after admission, but coincidentally, we found a middle cerebral bifurcation aneurysm when exploring the lateral fissure and then clipped it (Fig. 4). In the absence of CTA imaging, the evacuation of hematoma will be very passive for aneurysms found suddenly, which taught us a lesson. Fortunately, the aneurysms were not ruptured, so we clipped it safely. Obviously, this aneurysm had nothing to do with this injury. We speculate that this is not a traumatic aneurysm (TA), but an unruptured aneurysm. Fortunately, both bullets avoided this aneurysm. We suggest that, if time permits, head CTA should be performed in all patients regardless of whether such patients have a high risk of vascular injuries of the brain. It can not only help assess the vascular injury but also exclude vascular abnormalities of the patients themselves, which will play a key role in the final design of the operation.

Magnetic resonance imaging (MRI) studies are generally not recommended due to the uncertainty of bullet compositions. Moreover, in such an emergency, it is impractical to implement imaging modalities that are time-consuming to obtain [13].

### Surgical management

There is controversy regarding surgical management in patients with GWH. Most of the current treatment is based on data derived from retrospective observational military and civilian studies.

Minor gunshot injuries to the brain with non-penetrating wounds, such as tangential ricocheting wounds, may only require local debridement, closure, and antibiotics. More severe focal injuries with hemorrhage and fragments without a mass effect may also require local exploration via a small craniotomy. Tubular and penetrating wounds are still great challenges for neurosurgeons. Severe penetrating injuries will require extensive surgery, even if a decision is made to operate. This may include debridement, evacuation of hematomas, decompressive craniectomy, dural repair, and stereotaxic technique.

Recent evidence indicates that after satisfactory primary debridement, the risk of deep intracranial infections decreases sharply. In such circumstances, there is no need for secondary debridement. Long-term follow-up of such patients from the Vietnam, Iran-Iraq, and Israel-Lebanon Wars indicates that without complicating risk factors such as cerebrospinal fluid (CSF) leakage, the chance of intracranial infection does not increase [16]. When intractable intracranial hypertension (ICH) or malignant brain swelling is found at the time of a CT scan or surgical exploration, decompressive craniectomy could be life-saving. Experience during Operation Iraqi Freedom has indicated that decompressive craniectomy can offer an invaluable surgical technique for controlling intracranial pressure (ICP) or impending brainstem compression [17–21]. Decompressive craniectomy has been used in civilian GWH and seems to be of value when there is extensive hemispheric swelling that is not responsive to maximum medical management [21].

In several studies, patients underwent debridement and decompressive craniectomy. Kaufman et al. [8] reported 20 survivors and 8 non-survivors who had major debridement and decompressive craniectomy. In the study by Grahm et al. [6], 43% of patients underwent decompressive craniectomy specifically for debridement. Dosoglu et al. [6] reported 47 patients who underwent surgery, which included debridement and decompressive craniectomy. The concern in performing concurrent debridement and decompressive craniectomy is the potential development of cerebral abscesses [6, 22, 23]. Some authors have advocated a less aggressive cleaning procedure that preserves as much brain tissue as possible [24, 25], while others have suggested a more aggressive approach consisting of debridement of necrotic tissue, hematoma evacuation, removal of bone fragments, and foreign material as much as possible, establishing hemostasis, and dural closure [26–28]. Surgical intervention is not recommended for multilobular injuries and patients with a GCS score below 5 owing to a lack of survival benefit [6, 29]. Grahm et al. do not recommend surgery in the absence of any significant hematoma or a bihemispheric or multilobar injury, or when GCS score is above 6-8 [30]. The great challenges and dilemmas for neurosurgeons treating severe GWH are whether to pursue surgery and survival of the patient at all costs or whether to pursue quality of survival, so as to provide the expected treatment for selected patients [2]. In many cases, the results may not be easy to predict, which results in a difficult choice on behalf of surgeons in the choice of their treatment, but also largely subject to the willingness of patients’ families to trust the surgeon.

In our case, we decided to carry out the following operation plan: first, we quickly resected the temporal bone for decompression, then cut the dura mater to clear the wound along the trajectory and removed the temporal bullet and bone fragments. We did not aspirate much brain tissue, nor did we remove the occipital bullet fragment in the first operation. We aspirated the broken brain tissue and hematoma in the temporal lobe and maintained hemostasis, and at the same time, we clipped an unruptured aneurysm. This decision was dictated by several factors. The patient had a cerebral hernia at the time of admission (Fig. 1). The first problem to be solved is to relieve the compression of brain tissue. Decompressive craniectomy through the extended pterional approach can not only solve the problem of brain tissue compression but also remove the temporal bullet fragments and necrotic brain tissue. The reason why we did not remove the occipital bullet fragment at first is that the projectile trajectory in the occipital region was not clear and the extended pterional approach could not simultaneously remove the bullet fragment in the occipital lobe (Fig. 2). If the occipital bullet fragment was to be removed through a keyhole approach, we would have had to change the patient’s supine position to a lateral decubitus or prone position after the temporal surgery. This is a dramatic change in position, and it cannot guarantee the stability of the vital signs after the first surgery. Moreover, the occipital bullet fragment did not endanger life temporarily because it had no space-occupying effect. Finally, an unruptured aneurysm was found during the first operation. For the sake of caution, we decided to review CTA after the operation to determine the cerebrovascular condition. Therefore, we chose not to remove the occipital foreign body for the time being. After the patient’s vital signs were stable and CTA examination confirmed that there was no other vital vascular injury, the occipital bullet fragment was removed.

### Clinical management

ICH is a major prognostic predictor in patients with penetrating head trauma, with higher values associated with worse outcomes. ICH is the primary cause of mortality in patients with head trauma, and is a known contributor to secondary brain injury [31]. However, the need for ICP monitoring is not as well defined in the postoperative management of patients with civilian GWH in the management and prognosis of penetrating brain injury [3, 32, 33]. Commonly used methods for ICP correction are the infusion of hypertonic saline and mannitol, short-term hyperventilation, CSF drainage, barbiturates and paralytics, and finally decompressive craniectomy. Correction of ICP should be started at ICP values higher than the threshold of 20 mmHg registered for 5 min and longer.

Hyperventilation can be an efficient method for correcting ICH caused by cerebral hyperemia. When using hyperventilation, one should monitor whether oxygen supply to the brain is sufficient by measuring blood oxygen saturation in the jugular vein. Jugular venous oxygen saturation (SvjO_2_) indices lying within 55-75% are considered normal, provided that oxygenation of arterial blood is sufficient. The normal brain tissue oxygen (PbrO_2_) is 25-35 mmHg with arterial blood pressure at 80-100 mmHg. The partial pressure of CO_2_ (PaCO_2_) needs to be maintained at 36-40 mmHg. Mean blood pressure should be maintained above 90 mmHg during the entire duration of intensive care. However, preventative hyperventilation (PaCO_2_ < 35 mmHg) within the first 24 h has been shown to carry a risk of worsening cerebral perfusion by decreasing cerebral perfusion pressure [31].

Short-term hyperventilation is permitted in the case of abrupt worsening of neurological status or for persistently raised ICP despite the use of sedatives, muscle relaxants, hyperosmolar solutions, or CSF drainage [34, 35]. In our case, hyperventilation was a temporary measure for reducing increased ICP. Our patient underwent non-invasive ICP monitoring since the first operation and returned to the intensive care unit. In the next 48 h, he was administered continuous low-concentration oxygen therapy, and his GCS score reached 10 on the second day after surgery.

Civilian GWH are open, contaminated wounds that violate the scalp, skull, dura, and brain parenchyma. Devitalized brain tissue and retained bone fragments are suitable media for bacterial growth. Most contaminating organisms are those of skin flora, primarily *Staphylococcus epidermidis* [36, 37]. Patients with unknown immunization status and a contaminated wound to the head should receive a tetanus toxoid-containing vaccine intramuscularly and 250 U of human immunoglobulin against tetanus at a different site than the tetanus toxoid [38]. Although not supported by any prospective randomized controlled study, the use of prophylactic broad-spectrum antibiotics is appropriate for patients with penetrating brain injuries (PBI) since these wounds are considered to be contaminated [39]. According to the current US military guidelines, patients should receive cefazolin for 5-7 days [40]. Helling et al. stated that antibiotics, usually cephalosporins for blood-brain barrier penetration, were routinely administered to all patients, and no instances of postoperative brain abscesses have been reported [41, 42].

Seizure is another common complication seen in patients with traumatic brain injury, with an incidence ranging from 1.1-53%. Posttraumatic seizures are classified as early if the first seizure occurs within 7 days of the trauma, or late, if the first seizure develops 1 week after trauma [43–45]. Seizures were most often generalized with or without focal onset. Although no prospective study has indicated the efficacy of prophylactic antiseizure medications after a PBI, it has been recommended that patients be covered for about the first week after injury with a medication such as phenytoin or carbamazepine [46, 47]. Anticonvulsants such as phenytoin and carbamazepine are recommended for patients in the acute phase of severe traumatic brain injury with a high risk of seizure development. Results of class I studies show that preventative therapy with phenytoin, carbamazepine, phenobarbital, or valproate is not effective against late seizure onset in patients with traumatic brain injury [34].

### Complications

The implanted material along with necrotic brain and bone fragments are usually at the entrance of the incurred brain wound, and if the wound is not well-debrided, it can act as a site of infection [39]. Among the complications of GWH are CSF leaks or fistulas, which have been reported to occur in 0.63-8.9% of patients. They are more frequent in patients with ventricular involvement and incomplete dural and scalp closure, and the chance of infection was increased 20-fold in patients with CSF fistulas. The rate of Gram-negative infection was also 3 times higher in patients with CSF fistulas than in patients without CSF fistulas. Patients whose wounds are complicated by fistulas have more extensive wounds. If the ventricle is penetrated, CSF can leak into the missile tract into the subdural space and can leak through suture lines of the dural graft and skin. Aarabi recommended repeated lumbar puncture, spinal thecal drainage, or even insertion of a shunt in selected patients until the scalp suture line was healed completely [48]. In addition to the usual complications produced by penetrating head injuries, late-onset intracerebral hemorrhage caused by rupture of TAs has been a major cause of death. Early diagnosis of these vascular insults with prompt attention to a proper diagnostic and therapeutic protocol may prevent such potentially fatal events. The following are high risks of developing into TAs: passage of missile or bone fragments through areas of crowded vasculature and/or through the skull base (through Reil’s triangle or from one hemisphere to the other); a remarkable amount of hematoma within the entrance pathway that is visible on the predebridement CT scan; multiple shells or bone fragments scattered in paths that branch into various directions. The appropriate time for performing angiography to locate a TA is during the first 10 days after injury [49].

Angiography should be performed as soon as possible after encountering high-risk patients. Surgery to treat TAs is a difficult challenge because they usually do not have a neck suitable for clipping. TAs should either be excluded from the main circulation by trapping or they should be coagulated. In cases where the harboring vessel is a major artery, coating the aneurysm with muslin or fibrin glue or excision of the TA after extracranial-intracranial bypass might be the preferred method of surgery [50, 51]. In wars and in younger patients, Hunterian ligation of extracranial vessels harboring expansile and/or symptomatic TAs or arteriovenous fistulas is a very effective therapy and will usually not compromise cerebral blood perfusion [50].

## Conclusion

To summarize, GWH represent a high-mortality emergency for trauma surgeons. Aggressive management is essential to improve prognosis and patient outcomes. This case describes a patient who successfully recovered after two gunshots to the head. He underwent aggressive debridement and medical prophylaxis against seizures and infections. Given the severity of his injuries, he had a remarkable outcome and returned to his family and activities of daily living following a short hospital course.

## Data Availability

The data supporting the conclusions of this article are included within the article.

## Abbreviations

GWH: Gunshot wounds to the head
GCS: Glasgow Coma Scale
CT: Computed tomography
ATC: Acute traumatic coagulopathy
CTA: CT angiography
MRI: Magnetic resonance imaging
CSF: Cerebrospinal fluid
ICH: Intracranial hypertension
ICP: Intracranial pressure
TAs: Traumatic aneurysms
